# Combined Effects of PFAS and Metals on Cognitive Function

**DOI:** 10.3390/environments13060319

**Published:** 2026-06-07

**Authors:** Adeola Shogbaike, Emmanuel Obeng-Gyasi

**Affiliations:** 1 Department of Built Environment, North Carolina A&T State University, Greensboro, NC 27411, USA; 2 Environmental Health and Disease Laboratory, North Carolina A&T State University, Greensboro, NC 27411, USA

**Keywords:** PFAS, heavy metals, cognitive function, Bayesian kernel machine regression, environmental mixtures, CERAD, neurotoxicity

## Abstract

**Background:**

Heavy metals and per- and polyfluoroalkyl substances (PFAS) are widespread environmental pollutants that have been linked to worsening cognition, but how the two classes act together to shape cognitive function is still not well characterized. Drawing on data from the National Health and Nutrition Examination Survey (NHANES), this observational analysis evaluated how PFAS and metals are jointly related to performance across distinct cognitive domains in older adults.

**Methods:**

We analyzed 1447 adults aged 60 years and older from the 2011–2012 NHANES cycle in a cross-sectional design study. Metal levels in serum and whole blood were determined with standardized laboratory assays. Associations of single exposures and of the overall mixture with the CERAD word-learning and recall tasks, Animal Fluency, and the Digit Symbol Substitution Test were assessed using multivariable linear regression, together with Bayesian Kernel Machine Regression (BKMR).

**Results:**

Single-exposure models produced largely modest and inconsistent associations across the cognitive measures. Within the mixture models, PFAS, especially PFOA, PFDE, and PFOS, were repeatedly flagged as influential across several domains, whereas the metals tended to matter for specific outcomes only. The strongest negative signals at elevated joint exposure emerged for memory-related measures, notably CERAD Trials 1 and 2.

**Conclusions:**

Joint exposure to PFAS and heavy metals appears to influence cognitive domains unevenly, with memory-related measures seeming more responsive as combined exposure rises. These results reinforce the value of mixture-oriented analytic strategies when investigating environmental contaminants in relation to cognitive aging.

## 1. Introduction

Population aging across the globe has pushed dementia to the forefront of public health and social-care priorities, with worldwide cases anticipated to climb to 131 million by 2050 and the associated societal costs projected to surpass US $2 trillion by 2030 [1,2]. Within the United States, Alzheimer’s disease (AD) and related dementias are forecast to reach 14 million people by 2060, with women and racial/ethnic minorities [3]. Alzheimer’s Disease, the most common form of dementia, is becoming more prevalent as populations grow older, imposing heavy demands on patients, caregivers, and society [4]. Because available therapies largely target symptoms once the disease has developed, monitoring cognition and intervening early across the life course are essential to postpone onset and support healthy aging [5]. Pinpointing modifiable contributors to cognitive decline is therefore a public health priority.

Roughly 200 chemicals are recognized as neurotoxic to humans, yet only a small share of the more than 80,000 chemicals now in commercial use have been subjected to standardized developmental neurotoxicity testing [6–8]. Among the best-characterized environmental neurotoxicants are lead (Pb), cadmium (Cd), methylmercury (MeHg), and arsenic (As), which commonly occur alongside other persistent contaminants such as per- and polyfluoroalkyl substances (PFAS) in everyday exposure scenarios. Such exposures have been tied to multisystem toxicity and impaired cognition, even when exposure levels are comparatively low [9–11]. Combinations of metals can act additively, synergistically, antagonistically, or independently, with the potential to drive marked biochemical changes across brain regions. Yet the underlying mechanisms and health consequences of complex multi-metal exposures remain incompletely understood, especially beyond pairwise interactions [11].

Per- and polyfluoroalkyl substances (PFAS) are persistent man-made compounds that are gaining recognition as neurotoxicants [12] and have been linked to a broad spectrum of adverse health effects spanning the reproductive, immune, endocrine, hepatic, cardiovascular, and neurodevelopmental systems [13–27]. Owing to their robust carbon–fluorine bonds and their affinity for both water- and fat-soluble media, these compounds have seen extensive industrial and consumer use since the 1930s [28]. People are exposed mainly via contaminated food and drinking water, along with indoor dust, consumer goods, and workplace sources [29–31]. As a result, PFAS are pervasive, and detectable serum levels are present in roughly 97% of the U.S. population [32].

Metals and PFAS alike have been tied to biological processes underlying neurodegeneration and cognitive decline, such as oxidative stress, neuroinflammation, mitochondrial dysfunction, endocrine disruption, and disrupted neuronal signaling [33]. Certain metals—notably lead and mercury—can, additionally, perturb calcium homeostasis and weaken blood–brain barrier integrity, whereas PFAS have been reported to modify lipid metabolism and inflammatory signaling.

Epidemiological evidence suggests that prenatal PFAS exposure can hamper learning, memory, and intellectual development in children and raise the likelihood of behavioral conditions such as attention-deficit/hyperactivity disorder (ADHD) and autism spectrum disorder [30,34]. Such early-life and accumulated exposures may further add to cognitive decline over the life course, with possible effects on cognitive aging and dementia risk. However, the available human research on PFAS neurotoxicity and adult cognition is still sparse and inconsistent, with most studies centering on prenatal exposure and childhood neurodevelopment, rather than on combined environmental co-exposures across the life course [35–39]. The few adult-focused studies have produced mixed results: some point to apparently protective effects [40–42], whereas others describe harmful associations, for instance that of perfluorooctane sulfonate (PFOS), being tied to poorer cognitive scores among older adults with normal kidney function [43]. Newer findings suggest that low-dose exposure to PFAS mixtures may add to cognitive decline, with perfluorooctanoic acid (PFOA) appearing to play the largest part, although no meaningful PFAS interactions have been detected [44]. These studies consider single PFAS compounds on their own, and do not adequately address concurrent exposure to neurotoxic metals, potentially missing joint and interactive effects and understating the actual cognitive burden tied to everyday chemical mixtures [44].

Neuropsychological testing is central to detecting and tracking cognitive impairment early in mild cognitive impairment (MCI) and Alzheimer’s disease (AD) [45–47]. Such tests can reveal deficits years ahead of a clinical diagnosis, underscoring their sensitivity to early, subtle cognitive change [48–50]. They are especially useful for detecting environmentally driven cognitive effects that may emerge before clinical symptoms appear. Because conventional batteries are time-consuming and often impractical in large population-based studies, briefer standardized screening instruments have been developed that balance efficiency against sensitivity [51].

The Consortium to Establish a Registry for Alzheimer’s Disease neuropsychological assessment battery (CERAD-NAB) was created to meet this need, offering a standardized, efficient, and validated assessment of the cognitive domains most affected early in Alzheimer’s disease, namely verbal memory, fluency, and executive function [52,53]. Notably, CERAD indices—particularly delayed recall and learning efficiency—stay relatively stable in normal aging, yet decline early in pathological impairment, which makes them well-suited to environmental epidemiology aimed at separating age-related change from neurotoxicant-related decline [52,54].

Given the pervasive joint exposure to PFAS and neurotoxic metals, how the two jointly relate to cognitive function remains an important unanswered question in environmental health, especially in older adults, who may be more susceptible to environmental insults. The present study therefore set out to examine the combined associations of PFAS and metals exposure with domain-specific cognition, using CERAD-based neuropsychological measures in a nationally representative older-adult sample. We hypothesized that greater combined PFAS and metal exposure would be linked to poorer cognitive performance, particularly on memory-related tasks. By applying a mixture-based analytic framework to population-level data, the study seeks to clarify environmental contributions to cognitive aging and to inform prevention efforts and regulatory policy targeting modifiable environmental risk factors.

## 2. Materials and Methods

### 2.1 Study Design and Population

#### 2.1.1. Data Source

This work was a secondary analysis of publicly accessible data from the 2011–2012 cycle of the National Health and Nutrition Examination Survey (NHANES), a cross-sectional, nationally representative program run by the U.S. Centers for Disease Control and Prevention (CDC). NHANES uses a complex, multistage probability sampling scheme to characterize the health and nutritional status of the noninstitutionalized U.S. civilian population.

#### 2.1.2. Study Population

The analytic sample comprised 1447 participants aged 60 years and older drawn from the 2011–2012 NHANES cycle. To be included, participants had to have completed the cognitive assessments and to have valid blood-metal measurements (lead [Pb], cadmium [Cd], and mercury [Hg]), together with serum PFAS values (perfluorodecanoic acid [PFDE], perfluorooctanoic acid [PFOA], perfluorooctane sulfonic acid [PFOS], perfluorohexane sulfonic acid [PFHxS], perfluorononanoic acid [PFNA], and perfluoroundecanoic acid [PFUA]).

#### 2.1.3. Exclusion Criteria

Individuals were excluded when key covariates (demographic, socioeconomic, behavioral, or health-related) were missing or when biomarker values exceeded the 99.9th percentile, the latter serving to limit the impact of outliers. The CERAD assessment was not administered to participants who could not understand or read English, Spanish, Korean, Vietnamese, traditional or simplified Mandarin, or Cantonese, or who required a proxy informant.

### 2.2 Outcome Variable: Cognitive Function Assessment

Cognition was assessed within NHANES during household interviews and Mobile Examination Center (MEC) visits. The battery comprised the following: (1) the Word List Learning and Recall modules of the Consortium to Establish a Registry for Alzheimer’s disease (CERAD); (2) the Animal Fluency test; and (3) the Digit Symbol Substitution test. The CERAD Word List Learning subtest captures immediate and delayed acquisition of new verbal material [55]. This task comprises three successive learning trials (Trials 1–3) plus a delayed recall trial. Across the three learning trials, participants read aloud a list of 10 common, unrelated words, presented one at a time in a different random order on each trial [55]. Each trial is scored out of a maximum of 10. In NHANES, the learning-trial words appeared in large bold type on a computer screen, and participants who could not read them because of limited literacy or visual impairment instead repeated each word after the interviewer read it aloud.

The Animal Fluency test gauges categorical verbal fluency, an aspect of executive function [56]. It draws on general knowledge (e.g., naming animals) that does not depend heavily on the formal schooling of any particular culture [57]. Respondents name as many animals as they can within one minute, earning one point per animal. In NHANES, a practice task first asked them to name three articles of clothing, a separate fluency category; those unable to name three items of clothing did not proceed to the Animal Fluency task.

The Digit Symbol Substitution Test indexes processing speed, sustained attention, and working memory [58]. It uses a paper form whose header pairs nine numbers with nine symbols; within 2 min, participants fill the 133 boxes beneath the numbers with the matching symbols, and the score equals the number of correct entries. A short practice precedes the main test, and, in NHANES, those who could not complete the practice matches unaided did not continue.

The delayed Word List Recall followed the other two tasks (Animal Fluency and the Digit Symbol Substitution Test), roughly 8–10 min after the learning trials began, and required participants to recall the original 10 words. Alongside the scores for each learning trial and for delayed recall, the number of intrusions (words produced that were not on the list) was also recorded.

Every cognitive score was treated as a continuous outcome based on the recorded scores from the CERAD word-learning and delayed-recall tasks, the Animal Fluency test, and the Digit Symbol Substitution Test (DSST), where higher scores denote better cognitive performance. In practice, the analyses used the number of words recalled over the CERAD trials, the delayed-recall score, the count of animals named, and the DSST total. No fixed impairment threshold or categorical cutoff was imposed. The CERAD-NAB has been used in both cross-sectional and longitudinal work on dementia prevalence and incidence; beyond grading AD severity, it helps flag mild cognitive impairment, assist differential diagnosis, gauge the cognitive impact of environmental and experimental exposures, and illuminate brain–cognition relationships [59].

### 2.3 Predictor Variable: Exposure Assessment

#### 2.3.1. Metals

Whole-blood levels of lead (Pb), cadmium (Cd), and total mercury (Hg) were measured by inductively coupled plasma–mass spectrometry (ICP-MS) at the CDC’s National Center for Environmental Health. Concentrations falling below the limit of detection (LOD) were replaced with the LOD divided by the square root of 2 (LOD/√2).

#### 2.3.2. PFAS

Serum levels of the PFAS analytes—perfluorodecanoic acid (PFDE), perfluorooctanoic acid (PFOA), perfluorooctane sulfonic acid (PFOS), perfluorohexane sulfonic acid (PFHxS), perfluorononanoic acid (PFNA), and perfluoroundecanoic acid (PFUA)—were determined by online solid-phase extraction coupled with high-performance liquid chromatography–tandem mass spectrometry (HPLC–MS/MS), with values adjusted for serum sample weight. Readings below the limit of detection (LOD) were assigned the LOD divided by the square root of 2 (LOD/√2), to limit bias from left-censored data.

#### 2.3.3. Exposure Standardization

Prior to BKMR, all exposure variables—metals (Pb, Cd, and Hg) and PFAS (PFDE, PFOA, PFOS, PFHxS, PFNA, and PFUA)—were converted to z-scores, to improve comparability across the mixture components.

### 2.4 Covariates

Cognitive performance on the CERAD-based measures was the outcome. The predictors were the chemical exposures—metals (lead [Pb], cadmium [Cd], and mercury [Hg]) and PFAS (perfluorodecanoic acid [PFDE], perfluorooctanoic acid [PFOA], perfluorooctane sulfonic acid [PFOS], perfluorohexane sulfonic acid [PFHxS], perfluorononanoic acid [PFNA], and perfluoroundecanoic acid [PFUA])—chosen a priori from the literature. The models adjusted for demographic characteristics (age, sex, and race/ethnicity), socioeconomic indicators (education and household income), and behavioral and health factors (smoking and alcohol use). Smoking was defined as per the standard NHANES criterion of having smoked at least 100 cigarettes over the lifetime, and alcohol use as self-reported intake of at least 12 alcoholic drinks in a one-year span, in line with NHANES classifications widely used in epidemiologic work. These covariates were incorporated to address potential confounding and were harmonized following NHANES documentation.

### 2.5 Statistical Analysis

#### 2.5.1. Descriptive Statistics

Weighted summary statistics were used to characterize the sample and the distribution of each variable. Categorical variables (gender, education, and smoking status) were reported as frequencies and percentages, whereas continuous variables, including all CERAD scores, were reported as means and standard deviations. Before mixture modeling, Spearman coefficients were computed to examine pairwise correlations among the metals and PFAS. Weighted linear regression quantified the association of each individual exposure with each cognitive outcome, adjusting for all covariates, with results reported as β coefficients and 95% confidence intervals (CIs) per interquartile-range (IQR) increment in exposure. Descriptive and multivariable linear regression analyses were carried out in Stata SE version 19.5 (StataCorp, College Station, TX, USA), with statistical significance set at 0.05 for the non-Bayesian analyses; these regression models applied subsample weights and accounted for the complex NHANES survey design. Bayesian Kernel Machine Regression (BKMR) was then used to evaluate the combined and potentially interactive effects of the exposure mixture.

#### 2.5.2. Handling Missing Data

Missing values were handled with multiple imputation by chained equations (MICE) in R. The imputation model drew on the cognitive outcomes, the PFAS biomarkers (PFDE, PFOA, PFOS, PFHxS, PFNA, and PFUA), the metals (lead, cadmium, and mercury), and the relevant demographic, socioeconomic, and behavioral covariates (age, gender, ethnicity, education, income, smoking, and alcohol use). MICE automatically chose variable-specific conditional models, according to each variable’s data type. Fifty imputed datasets were created over 50 iterations under a fixed seed (seed = 123) for reproducibility; the number of imputations was set, to exceed the fraction of missing data and to stabilize the parameter estimates. The imputed data were then carried forward into the subsequent analyses, preserving sample size and reducing bias arising from missingness.

#### 2.5.3. Bayesian Kernel Machine Regression Analysis

Bayesian Kernel Machine Regression (BKMR) is being used more and more in environmental epidemiology to study the health effects of multi-pollutant mixtures, since it flexibly estimates exposure–response relationships that may be nonlinear or nonadditive [44]. All exposures were modeled jointly to recover nonlinear exposure–response surfaces and interactions, and posterior inclusion probabilities (PIPs) indexed each variable’s importance. A Gaussian predictor–response kernel was used with component-wise variable selection enabled. Each model was run as a single chain for 5000 Markov chain Monte Carlo (MCMC) iterations, discarding the first 1000 as burn-in and retaining the remainder for posterior inference, with a fixed seed for reproducibility. Convergence was checked by visually inspecting trace plots of the model parameters and the component-wise PIPs. All BKMR and Spearman analyses were run in R (version 4.5.1; R Foundation for Statistical Computing, Vienna, Austria). Because conventional BKMR cannot directly incorporate complex survey weights, these models were fitted without weighting and should be read as exploratory mixture analyses.

### 2.6 Ethical Considerations

The NHANES protocol received approval from the National Center for Health Statistics Research Ethics Review Board, and every participant gave written informed consent. As a secondary data analysis of de-identified, publicly available data, the present study was considered exempt from further institutional review.

## 3 Results

### 3.1 Descriptive Statistics

Table 1 reports descriptive statistics for the continuous variables—age, the metal exposures (lead, cadmium, and mercury), the PFAS exposures (PFDE, PFOA, PFHxS, PFOS, PFNA, and PFUA), and the cognitive tests (Trial 1, Trial 2, Trial 3, Animal Fluency Test, Digit Symbol Substitution Test, and Delayed Recall). Mean concentrations of lead, cadmium, and mercury were 1.81 μg/L, 0.54 μg/L, and 1.37 μg/L, respectively. The mean levels for PFDE, PFOA, PFHxS, PFNA, and PFUA were 0.31 μg/L, 3.17 μg/L, 2.56 μg/L, 1.35 μg/L, and 0.22 μg/L, respectively, while the mean level for PFOS was 12.72 μg/L. Participants had a mean age of 70.28 years.

Table 2 presents the categorical variables—gender, ethnicity, education, income, smoking status, and alcohol consumption—as frequencies and percentages. Gender was roughly balanced (48.86% male; 51.14% female). By race/ethnicity, 7.60% were Mexican American, 12.09% Other Hispanic, 40.98% Non-Hispanic White, 27.02% Non-Hispanic Black, 10.30% Non-Hispanic Asian, and 2.00% Other Race, including multiracial individuals. Education ranged widely: 18.73% had less than a 9th grade education, 16.17% had completed grades 9–11 (including 12th grade with no diploma), 23.91% were high school graduates or held a GED or equivalent, 25.22% reported a college or associate degree, and 15.82% were college graduates or above. A total of 50.21% of the participants reported having smoked at least 100 cigarettes in their lifetime, while 64.72% reported consuming at least 12 alcoholic drinks within one year.

### 3.2. Linear Regression

The linear regression results relating environmental exposures to the cognitive outcomes (CERAD Trials 1–3, Animal Fluency, Digit Symbol, and Delayed Recall) are shown in Table 3 as regression coefficients, standard errors, *p*-values, and 95% confidence intervals. For the most part, exposures were not significantly associated with the outcomes, though a few PFAS and metals displayed outcome-specific signals. Across CERAD Trials 1–3, the majority of exposures were unrelated to performance. In Trial 1, most chemicals, PFOA, PFHxS, PFNA, PFUA, and lead, were non-significant, with *p*-values above 0.05 and confidence intervals spanning zero, pointing to no meaningful linear association with CERAD scores in this model. PFDE was positively associated (β = 0.704, *p* = 0.011), whereas PFOS (β = −0.024, *p* < 0.001), cadmium (β = −0.272, *p* = 0.011), and mercury (β = −0.100, *p* = 0.000) were significantly negative. In Trial 2, no PFAS reached significance, but lead (β = −0.066, *p* = 0.043) and cadmium (β = −0.244, *p* = 0.021) were negatively associated. In Trial 3, none of the exposures were significant, although PFOS showed a borderline positive association (*p* = 0.074).

For Animal Fluency, several PFAS reached significance: PFDE (β = 1.983, *p* = 0.022), PFOA (β = 0.173, *p* = 0.023), and PFNA (β = 0.312, *p* < 0.001) were positively associated, whereas PFOS (β = −0.050, *p* = 0.001) and PFUA (β = −2.457, *p* = 0.002) were negatively associated. None of the metals were significant.

On the Digit Symbol Test, most exposures were non-significant; PFOA, however, was positively associated (β = 0.961, *p* < 0.001), and cadmium negatively associated (β = −2.031, *p* = 0.008). For Delayed Recall, several PFAS were significantly related to performance, while the metals largely were not.

In the Delayed Recall analysis, multiple PFAS were significant: PFDE, PFHxS, and PFNA were positively associated, while PFOA, PFOS, and PFUA were negatively associated. The metals were non-significant, though lead approached significance in the negative direction (*p* = 0.068). On balance, these results point to outcome-specific associations, with PFAS showing effects in both directions and metals showing weaker, less consistent relationships.

### 3.3. Spearman Correlation Matrix Analysis of PFAS and Metal Exposures

Relationships among the continuous variables were examined with a Spearman correlation matrix. As a nonparametric, rank-based measure, the Spearman coefficient captures the strength and direction of monotonic associations and is well-suited to data that do not satisfy the normality assumptions underlying Pearson correlation.

**Figure 1.**
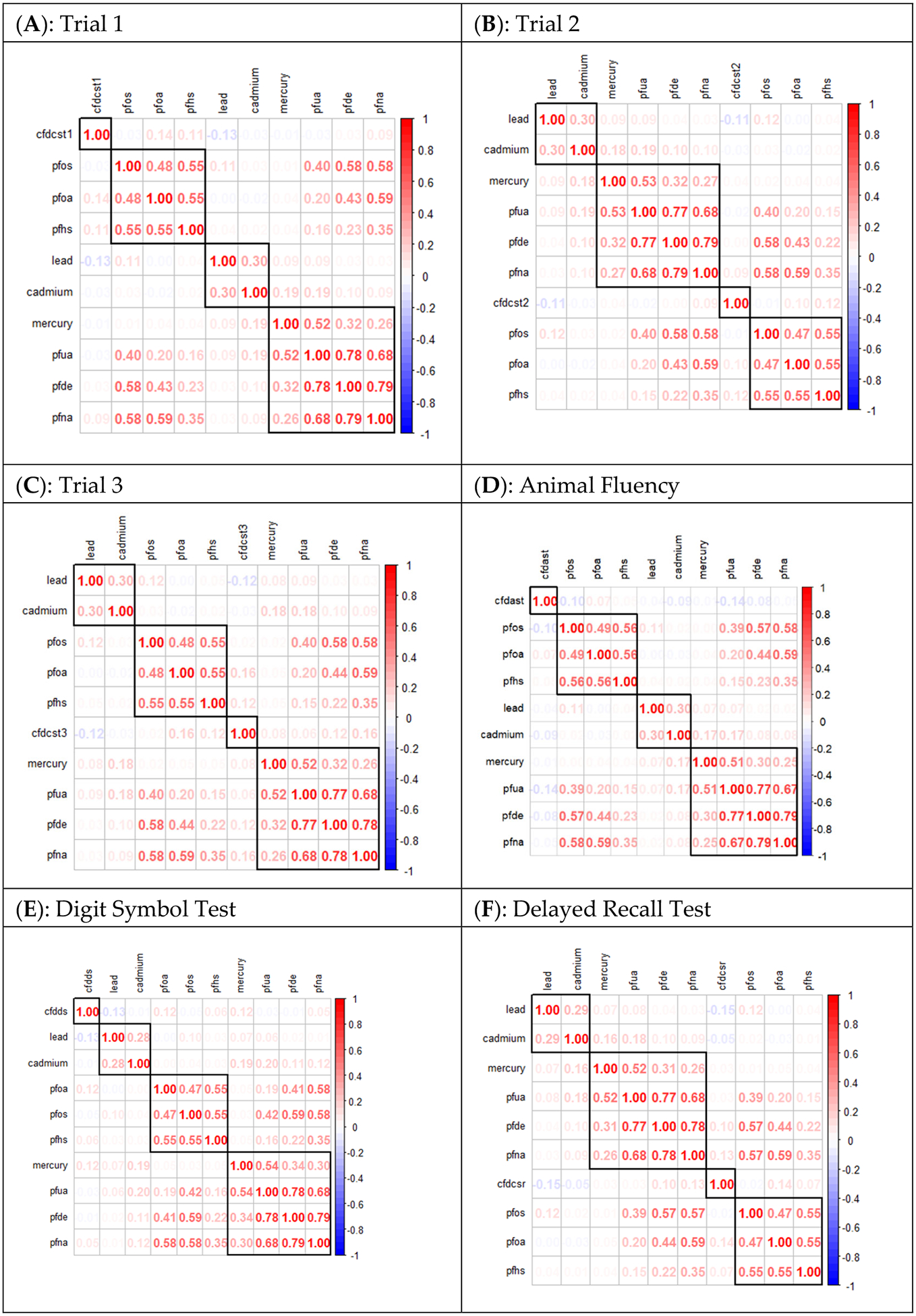
Correlation matrix of PFAS, metals, and CERAD cognitive function tests (Trials 1–3, Animal Fluency, Digit Symbol, Delayed Recall).

The Spearman correlation matrix among PFAS, metal exposures, and cognitive scores is shown in Figure 1.

Panels A–C (Trials 1–3) showed a consistent PFAS structure: short- to mid-chain PFAS (PFOS, PFOA, and PFHxS) were moderately intercorrelated (r ≈ 0.47–0.55) and long-chain PFAS (PFUA, PFDE, and PFNA), more strongly so (r ≈ 0.68–0.79). Correlations between metals and PFAS were generally weak, apart from a moderate mercury–PFUA correlation (r ≈ 0.51–0.53) and a modest lead–cadmium correlation (r ≈ 0.29–0.30). Across these trials, cognitive scores correlated minimally with the exposures (|r| ≤ 0.16). Panels D–E (Animal Fluency and Digit Symbol) showed comparable PFAS clustering and weak metal–PFAS links, with cognitive outcomes again barely correlated (|r| ≈ 0). Panel F (Delayed Recall) reproduced the same PFAS pattern, with mercury moderately tied to PFUA (r = 0.52) and lead modestly tied to cadmium (r = 0.29). Delayed Recall scores correlated weakly with every exposure, including a weak negative correlation with lead (r = −0.15) and weak positive correlations with PFOS and PFOA (r = 0.14 each). Taken together, these patterns reflect co-occurring exposures and describe the correlation structure of the PFAS–metal mixture ahead of mixture modeling.

### 3.4. Bayesian Kernel Machine Regression Analysis

#### 3.4.1. Insights from the Posterior Inclusion Probabilities

Within BKMR, the PIP reflects how likely each variable is to contribute to the model, and therefore serves as an index of variable importance.

Table 4 presents the PIPs estimated from BKMR for the environmental exposures assessed across the cognitive outcomes. Both PFAS and metals contributed to differences in cognitive performance, with the leading exposures differing by test. For word-list Trial 1, the principal contributors were PFOS, mercury, and PFDE; Trial 2 was dominated by cadmium and lead; and Trial 3 was shaped most by PFOS, PFDE, and PFOA. For the Animal Fluency Test, PFOA, PFUA, and PFOS were most influential, while for the Digit Symbol Test PFOA stood out as a highly relevant predictor. For the Delayed Recall Test, PFDE and PFOA were the most important exposures. Overall, select PFAS and, to a lesser extent, lead, emerged as relevant exposures in several cognitive domains, with importance varying by outcome rather than appearing uniformly across tests. These patterns underscore the value of mixture-based models for capturing the joint contributions of co-occurring exposures to neurocognitive performance.

#### 3.4.2. Univariate Exposure Response of PFAS and Metals on Cognitive Function

**Figure 2.**
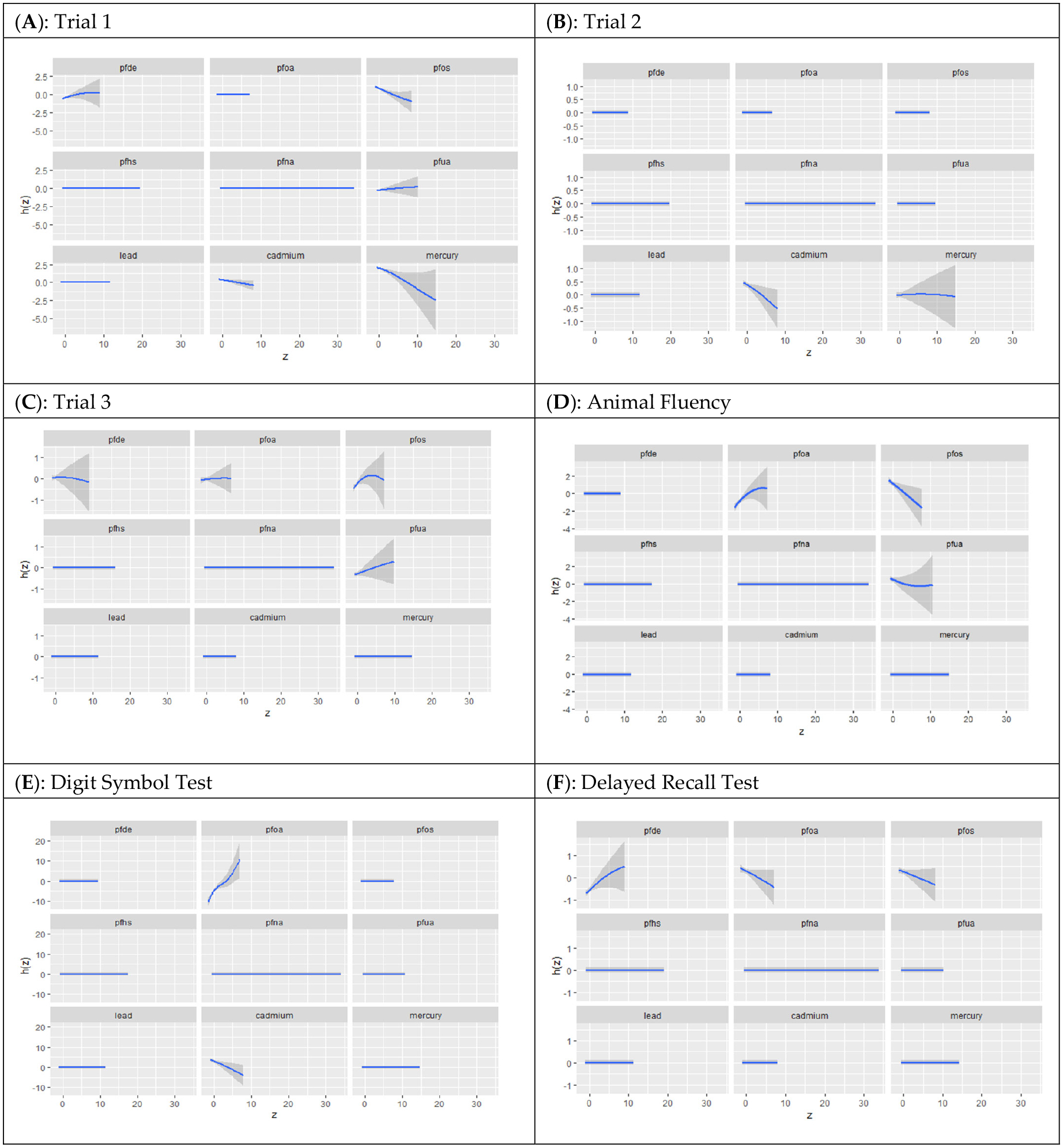
Panels (**A**–**F**). BKMR-derived univariate exposure–response curves for each environmental exposure, shown by the blue line, with the shaded gray region representing the corresponding 95% credible interval. Estimates were generated while holding the remaining PFAS and metal exposures at their median levels and adjusting for age, gender, smoking status, education, race/ethnicity, and income.

Figure 2 shows the BKMR-derived univariate exposure–response functions, with 95% credible intervals, for each individual exposure (PFDE, PFOA, PFOS, PFHxS, PFNA, and PFUA) and the metals (lead, cadmium, and mercury). Each curve reflects the posterior mean effect of one exposure, while the remaining mixture components are held at their medians, and the shaded band conveys the uncertainty around that relationship via the 95% credible interval. Exposure–response shapes differed across the six CERAD outcomes. In Learning Trial 1, PFOS, cadmium, and mercury trended negatively with performance, while PFDE and PFUA showed mild positive trends. In Trial 2, most PFAS hovered near zero, with broader but largely uninformative variability for cadmium and mercury. Trial 3 displayed modest non-linear shapes for PFDE, PFOS, and PFUA, with the other exposures flat. For Animal Fluency, PFOA was positive, while PFOS and PFUA were negative. In the Digit Symbol Test, PFOA trended strongly positive and cadmium negative. For Delayed Recall, PFDE was positive, whereas PFOA and PFOS trended negative, with the remaining exposures near null.

#### 3.4.3. Joint and Quantile-Based Bivariate Exposure Response

**Figure 3.**
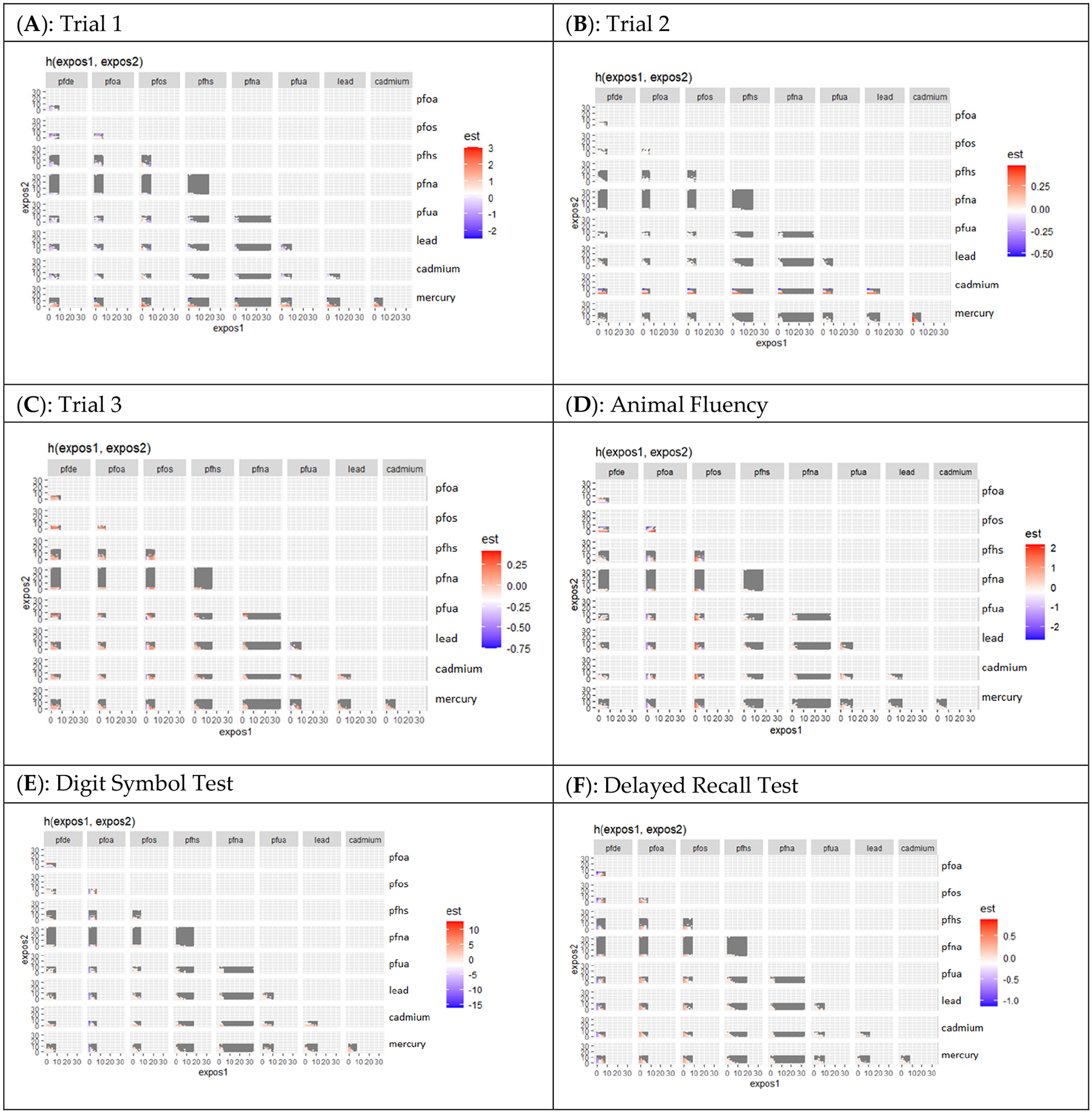
BKMR bivariate exposure–response function of PFAS and metals across six cognitive tests. Adjusted for gender, age, race/ethnicity, income, alcohol use, education and smoking status.

Figure 3 presents the BKMR bivariate exposure–response functions, depicting the joint effects of pairs of exposures—PFAS (PFDE, PFOA, PFOS, PFHxS, PFNA, and PFUA) and metals (lead, cadmium, and mercury)—on each cognitive outcome. It shows how varying two exposures together relates to cognition, while the remaining exposures stay at their medians. The color scale encodes the magnitude of the response, with red denoting positive and blue denoting negative associations, and gray marking regions of little evidence or greater uncertainty. Across the six CERAD outcomes, PFAS–PFAS and PFAS–metal interactions were generally small. In Trial 1 (Panel A), estimates clustered near zero, with only limited departures for PFDE, PFOS, and PFUA. Trial 2 (Panel B) was similar, with most interactions remaining near null aside, from modest localized shifts for PFOS, PFUA, and cadmium. In Trial 3 (Panel C), interactions were again largely minimal, with localized departures mainly in PFDE, PFOS, and cadmium pairings. For Animal Fluency (Panel D), interactions stayed mostly around zero, with modest shifts chiefly for PFDE, PFOS, PFUA, and cadmium. For the Digit Symbol Test (Panel E), interactions were weak overall, though somewhat stronger positive or negative shifts appeared for combinations of PFDE, PFOS, PFUA, and cadmium. For the Delayed Recall Test (Panel F), interaction patterns were similarly limited, with small localized departures mainly for PFOS, PFUA, and cadmium.

#### 3.4.4. Bivariate Exposure Response with Quantiles for Second Exposure

**Figure 4.**
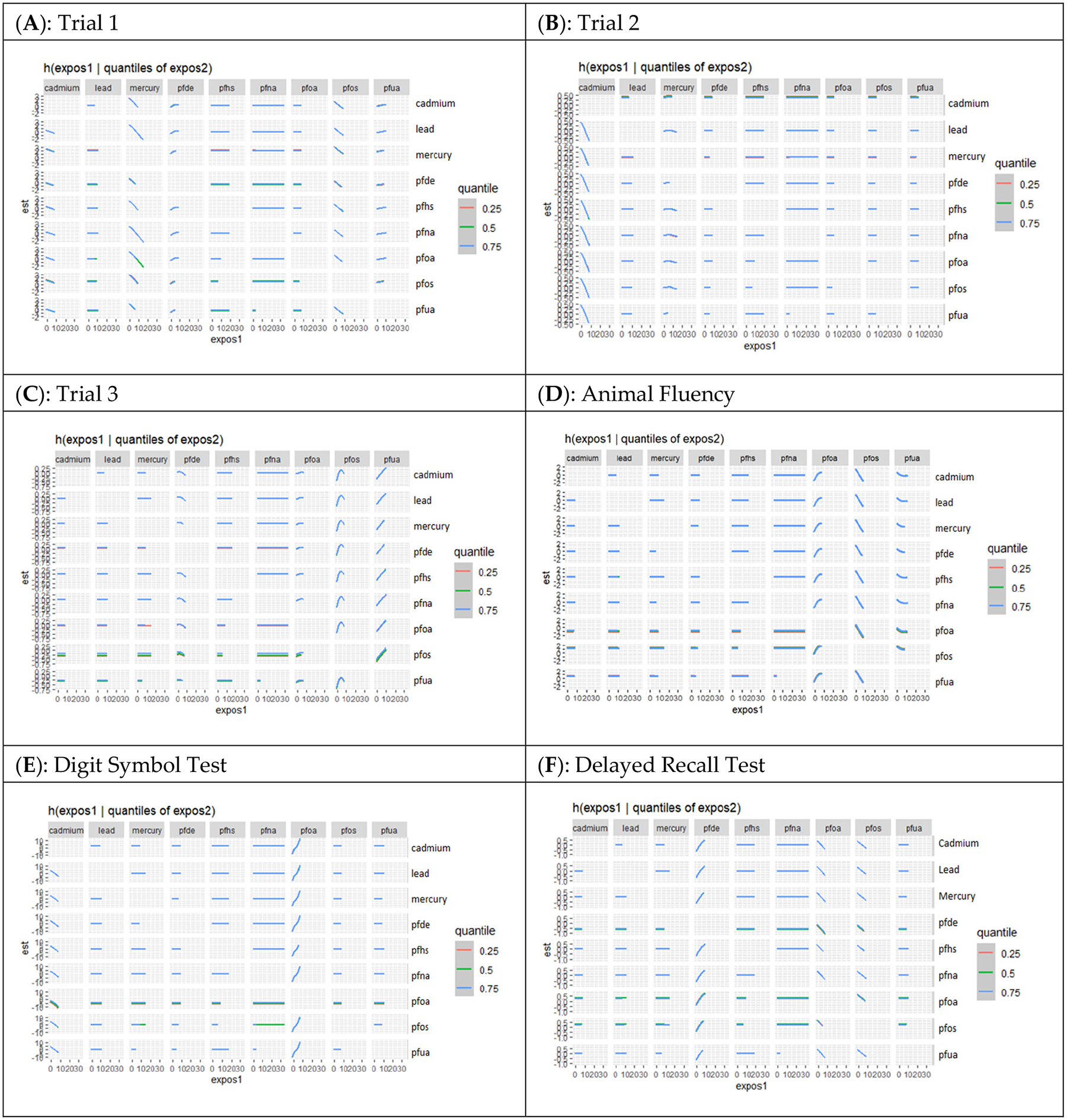
BKMR-estimated conditional bivariate exposure–response functions illustrating how the association between one exposure (Expos1) and cognitive outcomes varies across quantiles (0.25, 0.50, and 0.75) of a second exposure (Expos2), with all remaining components of the chemical mixture fixed at their median values. Lines represent posterior mean estimates, and color denotes the quantile of Expos2. Models were adjusted for age, gender, race/ethnicity, income, alcohol use, smoking status, and education.

Figure 4 shows the BKMR bivariate exposure–response functions, illustrating how the estimated effect of one exposure (expos1) on cognition shifts across levels of a second exposure (expos2). Expos2 was set at its 0.25, 0.50, and 0.75 quantiles to represent low, medium, and high co-exposure. These trajectories indicate whether the influence of expos1 changes as expos2 rises, offering a window into possible effect modification within the mixture. For Trials 1–3, conditional effects for most PFAS–PFAS and PFAS–metal pairs sat near zero, with modest quantile-dependent shifts mainly for PFDE, PFOS, and PFUA. The Animal Fluency and Digit Symbol tests behaved similarly, with associations largely flat across quantiles aside from small deviations involving PFDE, PFOS, and PFUA. For the Delayed Recall Test, most associations again centered near zero, the clearest quantile-related differences appearing for PFDE, PFOS, and PFOA.

#### 3.4.5. Overall Exposure Effect

**Figure 5.**
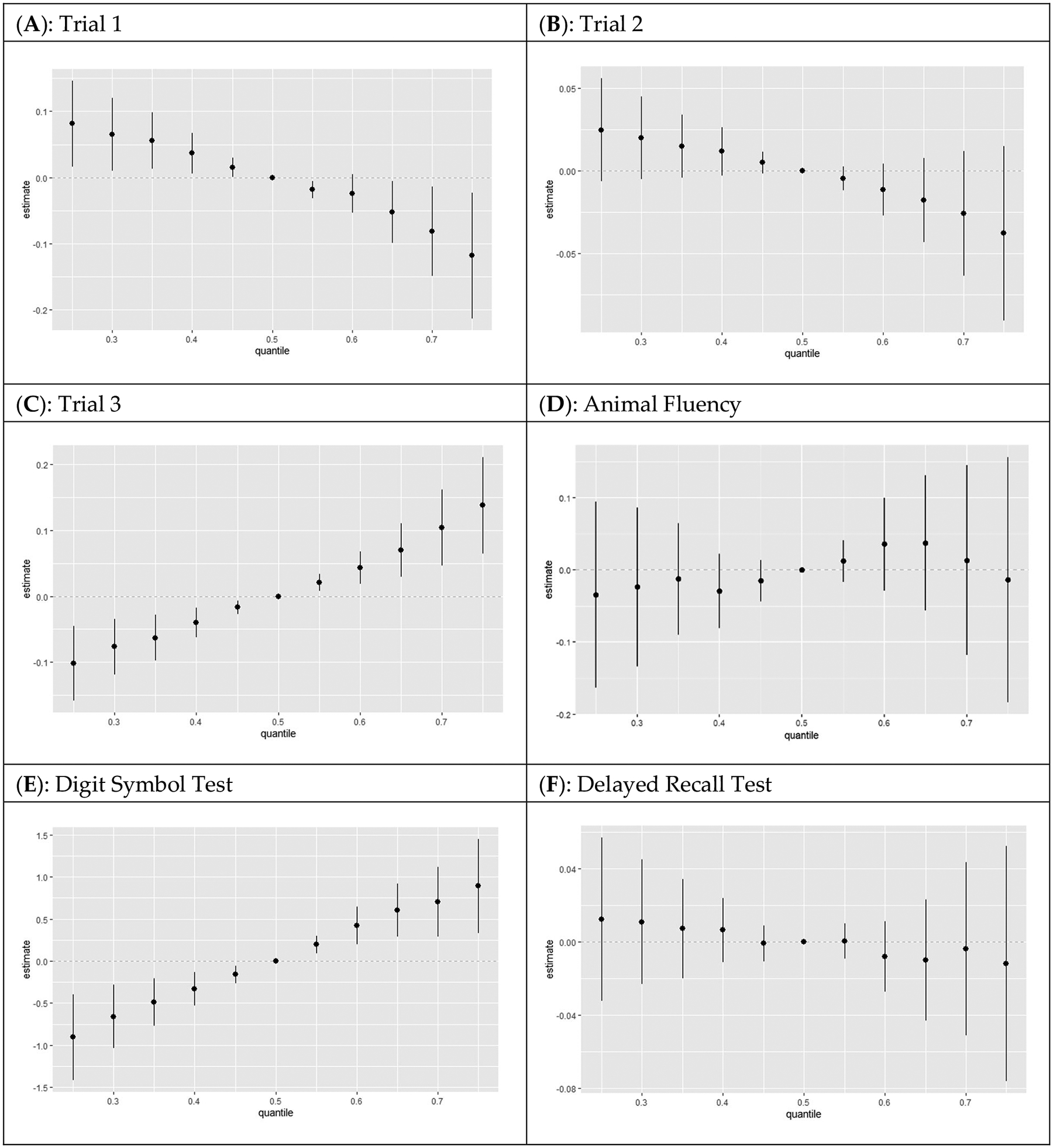
BKMR-derived exposure–response contrasts for individual exposures across the 0.25 to 0.75 quantile range, using the median exposure level, the 0.50 quantile, as the reference. Each point shows the posterior mean estimate, while the vertical bars indicate the associated 95% credible intervals.

Figure 5 summarizes the BKMR-estimated overall mixture effect on each cognitive outcome across quantiles from 0.25 to 0.75. Each point is the posterior mean contrast at a given exposure quantile, and the vertical lines give the corresponding 95% credible intervals. These conditional effects varied across quantiles of the joint exposure. For both Trial 1 and Trial 2, the lower quantiles (0.25–0.45) yielded mostly positive posterior means, though the credible intervals largely overlapped with zero, signaling considerable uncertainty. Near the median (0.50 quantile), the estimate sat close to zero, indicating little departure from the null at the midpoint. At the upper quantiles (0.55–0.75), the posterior means turned progressively negative, with larger effect sizes as the quantile rose. Even with persistent uncertainty—credible intervals frequently spanning zero—this downward shift at the upper quantiles is consistent with stronger negative associations between the mixture and cognition among people with higher joint exposure. The was pattern reversed for Trial 3 and the Digit Symbol Test: posterior means were mostly negative at the lower quantiles (0.25–0.45), centered near zero at the median (0.50 quantile), and increasingly positive at the upper quantiles (0.55–0.75), with effect sizes growing as the quantile rose, suggesting a strengthening association at higher co-exposure. For Animal Fluency, posterior estimates stayed near zero across quantiles with wide credible intervals, showing no consistent conditional mixture effect. The Delayed Recall Test likewise varied little across quantiles, with posterior means close to zero throughout. Although uncertainty persists—again with credible intervals that often overlap with zero—the cross-quantile trends hint at possible variation in the direction and size of mixture effects, particularly at higher exposure, and should be read with caution.

#### 3.4.6. Single/Exposure Effect Analysis

**Figure 6.**
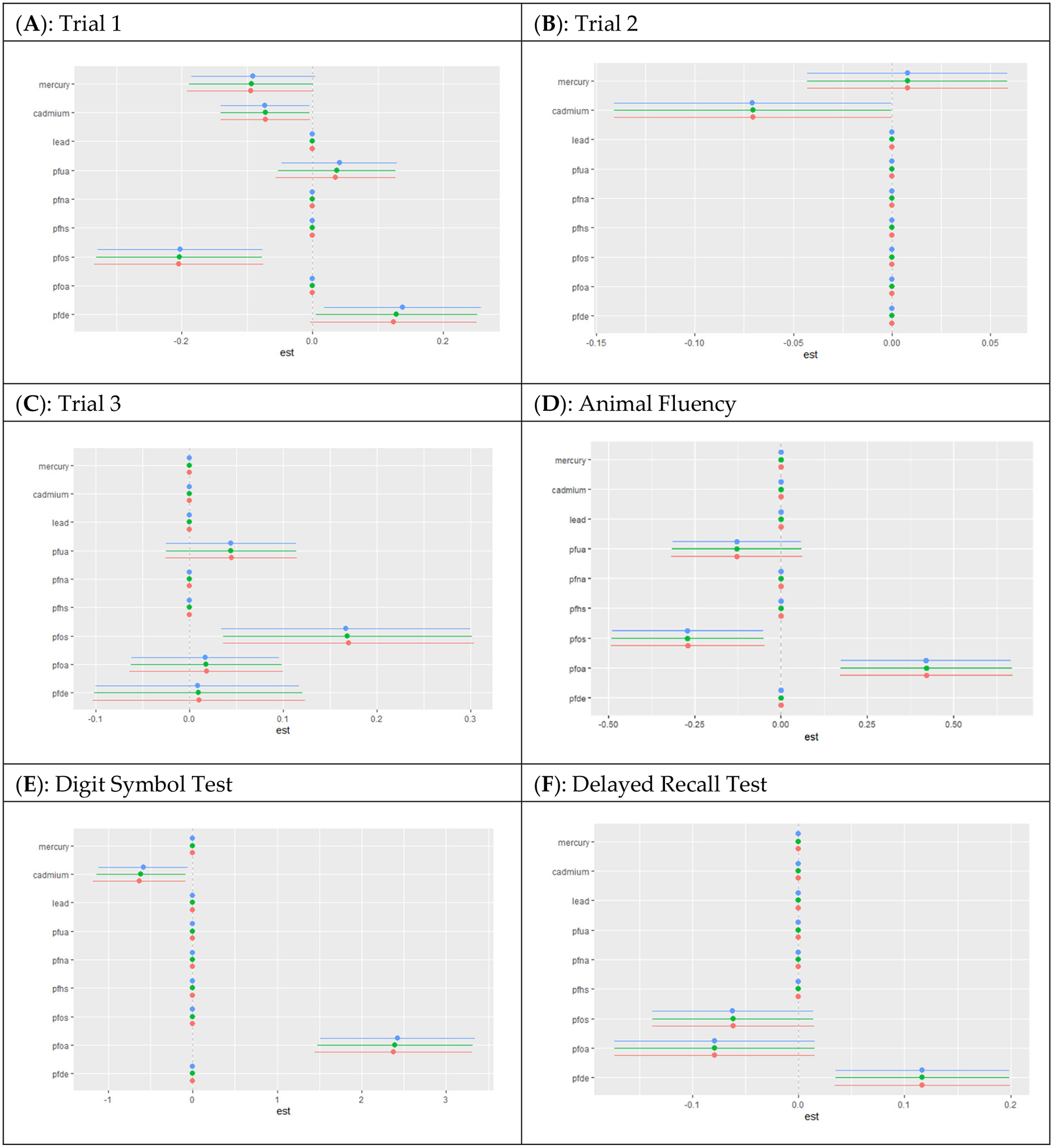
BKMR-derived single-exposure estimates showing the association between each environmental chemical and cognitive function while all other mixture components were held constant at the 0.25, 0.50, or 0.75 quantile. Points represent posterior mean estimates for each chemical, and horizontal bars show the corresponding 95% credible intervals.

Figure 6 shows the BKMR single-exposure functions for cognition by estimating how the response changes as each exposure increases from the 0.25 to the 0.75 quantile, while the remaining mixture components are held fixed at the 0.25, 0.50, or 0.75 quantile. The exposures examined included the metals mercury, cadmium, and lead, as well as the PFAS compounds PFUA, PFNA, PFHxS, PFOS, PFOA, and PFDE. For each chemical, posterior mean effects under the three co-exposure settings are plotted as color-coded points (blue for 0.25, green for 0.50, and red for 0.75), with horizontal lines marking the 95% credible intervals. The figure underscores how a single exposure’s estimated effect depended on the surrounding mixture. In Trial 1, PFDE had the strongest positive estimates and PFOS the negative ones, while most others stayed near zero. In Trial 2, nearly all exposures clustered around zero, with cadmium clearly negative. In Trial 3, PFOS showed the strongest positive effect. Animal Fluency and the Digit Symbol Test behaved similarly, with PFOA positive and cadmium negative. For Delayed Recall, PFDE was positive, though its wide credible interval signals appreciable uncertainty.

#### 3.4.7. Single-Variable Interaction Effect

**Figure 7.**
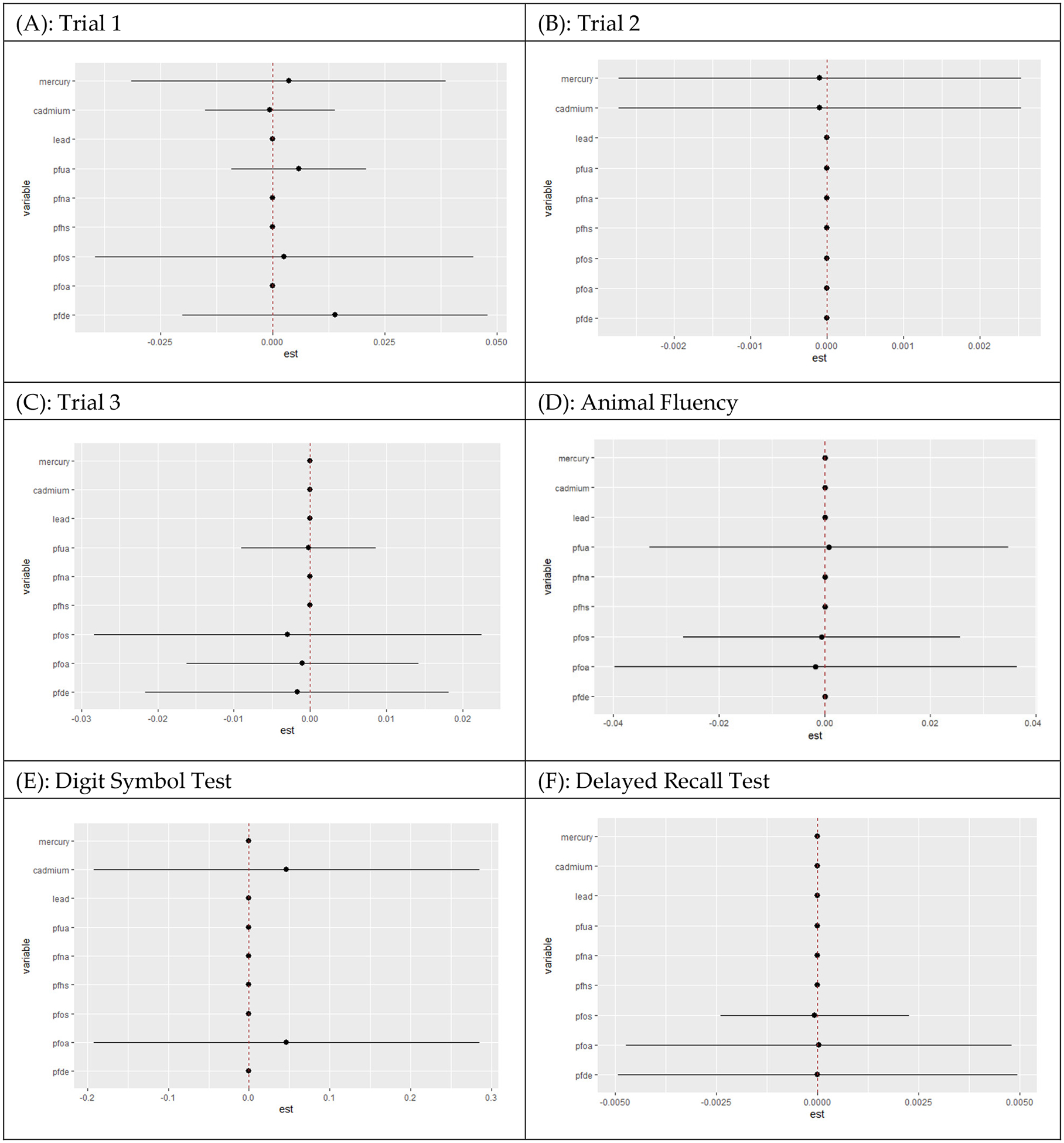
BKMR-derived estimates of single-variable interaction effects for the environmental exposure mixture. For each exposure, the plotted point and interval summarize the change in the estimated effect when that exposure is shifted from the 0.25 to the 0.75 quantile while all other exposures are fixed at either the 0.25 or 0.75 quantile. Black points represent posterior mean estimates, and horizontal bars indicate the corresponding 95% credible intervals.

Figure 7 displays the BKMR single-variable interaction functions for all exposures. For each chemical, the posterior mean contrast is plotted on the *x*-axis with its 95% credible interval as a horizontal line; black points mark the posterior means, and the vertical red line at zero denotes no departure from the null. These summaries describe how the effect of moving an exposure from its 0.25 to its 0.75 quantile changes when the rest of the mixture is held at either the lower or the upper quantile. In Trial 1, PFOS, PFDE, PFUA, and mercury showed modest positive estimates, whereas cadmium leaned slightly negative. In Trial 2, mercury and cadmium leaned negative, while in Trial 3, PFUA, PFOS, PFOA, and PFDE showed negative estimates with wide credible intervals, underscoring uncertainty. For Animal Fluency, PFUA, PFOS, and PFOA varied most, while the metals stayed tightly around zero. For Digit Symbol, cadmium and PFOA were slightly positive and the rest near zero; for Delayed Recall, the PFAS (PFDE, PFOA, and PFOS) again varied most, with the widest credible intervals indicating uncertainty.

## 4. Discussion

The present study evaluated the joint associations of PFAS and metal exposures with several cognitive domains in a nationally representative sample of older U.S. adults. On the whole, the associations between exposures and cognition were modest, heterogeneous, and domain-specific, with little sign of consistent linear relationships across the measures. Observed PFAS and metal levels were broadly in line with values reported for the general U.S. adult population in NHANES-based exposure surveillance, indicating that the exposures here reflect typical background levels, rather than acutely elevated or occupational ones. Few enforceable blood- or serum-based regulatory thresholds exist for these biomarkers, and where guidance does exist—such as drinking-water limits for certain PFAS—it pertains to environmental media, rather than internal dose, which limits direct comparison. These observations align with earlier environmental research indicating that PFAS and metal exposures may follow complex, non-monotonic dose–response patterns [43,60]. Although most single-exposure associations were small and inconsistent, a few PFAS—particularly PFOA, PFOS, and PFDE—recurred across the mixture analyses, hinting at possible relevance to cognition in aging populations. Across CERAD Trials 1–3, which assess immediate learning and memory processes, associations were generally weak and inconsistent, with only a handful of significant results. This accords with earlier NHANES-based work showing that PFAS–cognition relationships are frequently non-linear, small in magnitude, and sensitive to confounders such as kidney function and diet [43]. Within Trial 1, PFDE was positively and PFOS negatively associated, in line with earlier reports [43] and reflecting the compound-specific, heterogeneous character of environmental mixtures. In Trial 2, lead and cadmium were negatively related to performance, while Trial 3 yielded no significant associations. The negative findings for lead, cadmium, and mercury fit the well-documented neurotoxicity reported in epidemiologic and mechanistic work [6,61–63]. By contrast, stronger and more variable associations emerged for the domain-specific outcomes. On Animal Fluency, PFDE and PFNA were positive, while PFOS and PFUA were negative. On the Digit Symbol Test, PFOA was positively associated, echoing the findings of Weng et al., whereas cadmium was negative. The clearest pattern appeared for Delayed Recall, where several PFAS were significant in opposing directions, while the metals were mostly non-significant. These results imply that environmental exposures may act on particular cognitive domains, rather than uniformly on global cognition, with learning and memory perhaps being more vulnerable [43]. However, the inconsistent direction and magnitude of associations across cognitive tests also highlight the complexity of cognitive aging and the challenges of detecting subtle environmental effects in cross-sectional population studies.

The heterogeneity seen across PFAS compounds may reflect differences in chemical structure, toxicokinetics, biological mechanisms, and exposure pathways. Experimental and epidemiologic evidence indicates that PFAS can traverse the blood–brain barrier, build up in brain tissue [64], and interfere with key neurobiological processes such as calcium signaling, synaptic transmission, and neuronal plasticity, which are essential for learning and memory [65]. Conversely, the positive associations for PFDE, PFHxS, and PFNA may reflect dose-dependent or non-monotonic behavior, in keeping with prior reports of non-linear PFAS–cognition relationships in population studies [43]. The negative cadmium and lead associations in select outcomes agree with earlier evidence of metal-related cognitive impairment, possibly mediated by oxidative stress and neuronal injury [62], although the overall patterns in this study were limited and inconsistent. Simultaneous exposure to PFAS and metals could thus yield additive or synergistic neurotoxicity via shared biological pathways. Even so, the mechanisms behind combined PFAS–metal neurotoxicity are still poorly characterized, especially in humans exposed to low-dose environmental mixtures.

Although individual-exposure associations were generally modest and came with considerable uncertainty, the mixture analyses pointed to heterogeneous, non-linear exposure–response patterns across domains. PFAS were flagged more consistently than metals in the mixture models, especially for memory-related outcomes, whereas the metals showed more outcome-specific associations. This may indicate that PFAS bear more broadly on cognitive variability across domains, though the estimates were imprecise and warrant cautious interpretation. This is consistent with the regression results, and suggests that some PFAS may be relevant to cognition across multiple domains [66], while metals appear to act more selectively.

Notably, the BKMR analyses did not single out any one exposure as having a strong, consistent independent effect across all outcomes. Rather, they are consistent with cognitive effects arising from the cumulative or interactive influence of multiple co-occurring contaminants instead of single chemicals—particularly pertinent because everyday exposures seldom occur in isolation. The non-linear trends across exposure quantiles further underscore the need to analyze mixtures with flexible methods able to capture complex exposure–response relationships. The several inverse or apparently protective associations seen here also warrant caution. They may stem from residual confounding, differences in exposure source, reverse causation, or non-linear exposure–response relationships, rather than genuine protective biology. For instance, higher mercury may track with a greater fish intake, which supplies nutrients such as omega-3 fatty acids that can benefit cognition. Likewise, socioeconomic status, diet, healthcare access, and lifestyle may shape both exposure and cognitive outcomes. Although the models were adjusted for the main demographic and behavioral covariates, residual confounding from unmeasured or imperfectly measured factors cannot be ruled out.

Collectively, these findings highlight the importance of studying environmental exposures as mixtures, rather than as isolated compounds. The variation in direction, magnitude, and domain specificity suggests that the exposure–cognition relationship may be complex, non-linear, and potentially interactive. They also speak to the usefulness of flexible approaches such as BKMR for capturing the multidimensional nature of everyday exposures and for advancing understanding of the environmental determinants of cognitive health.

The study also has clear strengths: it examined PFAS and metals together, drew on several domain-specific cognitive outcomes, and applied Bayesian Kernel Machine Regression to capture non-linear and interactive exposure–response relationships that conventional linear models cannot.

### 4.1. Limitations

A number of limitations should be noted. First, the cross-sectional design constrains causal inference, since exposures and outcomes were captured at a single time point. Second, several estimates had wide uncertainty intervals, so larger and more diverse samples are needed to confirm the key findings. The cognitive battery, moreover, taps specific domains and may not fully represent broader cognitive function. Longitudinal studies with repeated biomarker sampling, clinical evaluation, and mechanistic experiments will be needed to firm up causal interpretation and to clarify dose–response relationships. Finally, because the BKMR models did not use NHANES survey weights, those mixture results are exploratory, and may not be nationally representative; the weighted regression analyses therefore carry the primary population-level inference, with BKMR providing complementary insight into mixture patterns. We also did not apply a formal multiple-comparisons correction; given the many exposures and outcomes assessed, some individual associations could be chance findings, so interpretation emphasized consistency across analyses and biological plausibility over isolated statistical significance.

### 4.2. Public Health Implications

These results carry several implications for public health practice and environmental policy. First, the associations between certain PFAS—notably PFOA, PFDE, and PFOS—and the cognitive outcomes suggest these exposures may contribute modestly to differences in cognitive function. Although the effect sizes are generally small, they may matter, given how widespread and chronic these exposures are. Second, the domain-specific, non-linear patterns and the evidence of mixture effects expose the limits of single-chemical risk assessment and argue for cumulative, mixture-based frameworks in environmental health research. Finally, the variation across cognitive domains suggests that future studies and surveillance may gain from assessing several facets of cognition, rather than a single global score, especially in exposed populations.

## 5 Conclusions

In sum, joint PFAS and metal exposure showed modest, heterogeneous, and domain-specific associations with cognition in older U.S. adults. Memory-related domains seemed more sensitive to higher combined exposure, and PFAS were associated more broadly across outcomes than the metals. Although the associations were modest and uncertain, the results emphasize the importance of considering environmental mixtures in studies of cognitive aging, and lay the groundwork for future longitudinal and mechanistic work on the long-term cognitive effects of combined environmental exposures.

## Figures and Tables

**Table 1.** Weighted descriptive characteristics of the study population.

	Mean	Standard Error	[95% Conf. Interval]	
Age (years)				
age	70.28	0.40	69.44	71.12
Metal exposures				
Lead	1.81	0.05	1.70	1.92
Cadmium	0.54	0.02	0.50	0.57
Mercury	1.37	0.13	1.10	1.64
PFAS exposures				
PFDE	0.31	0.03	0.25	0.36
PFOA	3.17	0.22	2.71	3.64
PFOS	12.72	0.75	11.13	14.31
PFHxS	2.56	0.39	1.74	3.37
PFNA	1.35	0.12	1.09	1.61
PFUA	0.22	0.03	0.17	0.28
Cognitive function scores				
Trial 1	4.67	0.11	4.44	4.91
Trial 2	6.55	0.10	6.35	6.75
Trial 3	7.35	0.09	7.16	7.53
Animal Fluency	17.73	0.30	17.09	18.36
Digit Symbol	50.25	1.09	47.95	52.54
Delayed Recall	5.74	0.12	5.48	6.00

**Table 2.** Survey-weighted characteristics of the study population, including demographic and behavioral factors.

Variable	Category	Frequency	Percent
Gender	Male	707	48.86
	Female	740	51.14
Ethnicity/Race	Mexican American	110	7.602
	Other Hispanic	175	12.09
	Non-Hispanic White	593	40.98
	Non-Hispanic Black	391	27.02
	Non-Hispanic Asian	149	10.30
	Other Race—Including Multi-Racial	29	2.004
Education	Less than 9th grade	271	18.73
	9–11th grade (includes 12th grade with no diploma)	234	16.17
	High school graduate/GED or equivalent	346	23.91
	Some college or AA degree	365	25.22
	College graduate or above	229	15.83
Income	$0 to $4999	29	2.004
	$5000 to $9999	111	7.671
	$10,000 to $14,999	198	13.68
	$15,000 to $19,999	150	10.37
	$20,000 to $24,999	138	9.537
	$25,000 to $34,999	207	14.31
	$35,000 to $44,999	163	11.26
	$45,000 to $54,999	97	6.704
	$55,000 to $64,999	96	6.634
	$65,000 to $74,999	70	4.838
	$75,000 to $99,999	112	7.740
Smoking	Yes	725	50.21
	No	719	49.79
Alcohol	Yes	822	64.72
	No	448	35.28

**Table 3.** Survey-weighted associations between PFAS, metals, and cognitive function outcomes.

A: Trial 1					B: Trial 2				
Variable	Coef	Std. Err.	Pr(>|t|)	95% Conf. Interval	Variable	Coef	Std. Err.	Pr(>|t|)	95% Conf. Interval
PFDE	0.704	0.276	0.011	(0.163, 1.245)	PFDE	−0.080	0.297	0.789	(−0.663, 0.504)
PFOA	−0.018	0.028	0.512	(−0.072, 0.036)	PFOA	0.009	0.027	0.742	(−0.045, 0.063)
PFOS	−0.024	0.006	0.000	(−0.035, −0.012)	PFOS	0.000	0.006	0.946	(−0.012, 0.011)
PFHxS	0.033	0.022	0.137	(−0.01, 0.076)	PFHxS	−0.035	0.023	0.122	(−0.08, 0.009)
PFNA	0.006	0.021	0.768	(−0.035, 0.047)	PFNA	−0.025	0.021	0.241	(−0.066, 0.017)
PFUA	0.237	0.259	0.361	(−0.272, 0.745)	PFUA	0.427	0.264	0.107	(−0.092, 0.945)
Lead	−0.010	0.031	0.744	(−0.072, 0.051)	Lead	−0.066	0.032	0.043	(−0.129, −0.002)
Cadmium	−0.272	0.107	0.011	(−0.481, −0.063)	Cadmium	−0.244	0.106	0.021	(−0.452, −0.037)
Mercury	−0.100	0.026	0.000	(−0.151, −0.049)	Mercury	−0.026	0.027	0.328	(−0.079, 0.026)
C: Trial 3					D: Animal Fluency				
Variable	Coef	Std. Err.	Pr(>|t|)	95% Conf. Interval	Variable	Coef	Std. Err.	Pr(>|t|)	95% Conf. Interval
PFDE	−0.070	0.281	0.803	(−0.621, 0.481)	PFDE	1.983	0.865	0.022	(0.287, 3.678)
PFOA	0.009	0.027	0.725	(−0.043, 0.062)	PFOA	0.173	0.076	0.023	(0.023, 0.322)
PFOS	0.010	0.005	0.074	(−0.001, 0.021)	PFOS	−0.050	0.015	0.001	(−0.08, −0.02)
PFHxS	−0.004	0.019	0.820	(−0.041, 0.033)	PFHxS	0.016	0.055	0.771	(−0.093, 0.125)
PFNA	−0.004	0.021	0.850	(−0.044, 0.037)	PFNA	0.312	0.058	0.000	(0.197, 0.427)
PFUA	0.284	0.251	0.256	(−0.207, 0.776)	PFUA	−2.457	0.798	0.002	(−4.023, −0.891)
Lead	−0.026	0.031	0.410	(−0.087, 0.035)	Lead	−0.044	0.089	0.620	(−0.219, 0.131)
Cadmium	0.048	0.104	0.642	(−0.155, 0.252)	Cadmium	−0.319	0.297	0.282	(−0.901, 0.263)
Mercury	0.002	0.026	0.929	(−0.049, 0.054)	Mercury	0.032	0.071	0.655	(−0.108, 0.171)
E: Digit Symbol Test					F: Delayed Recall Test				
Variable	Coef	Std. Err.	Pr(>|t|)	95% Conf. Interval	Variable	Coef	Std. Err.	Pr(>|t|)	95% Conf. Interval
PFDE	−0.665	2.184	0.761	(−4.948, 3.619)	PFDE	2.006	0.348	0.000	(1.323, 2.69)
PFOA	0.961	0.203	0.000	(0.562, 1.359)	PFOA	−0.092	0.036	0.011	(−0.163, −0.021)
PFOS	0.037	0.040	0.357	(−0.042, 0.116)	PFOS	−0.024	0.007	0.001	(−0.038, −0.01)
PFHxS	0.179	0.150	0.235	(−0.116, 0.473)	PFHxS	0.057	0.028	0.046	(0.001, 0.112)
PFNA	0.064	0.155	0.678	(−0.239, 0.368)	PFNA	0.058	0.026	0.028	(0.006, 0.11)
PFUA	−0.210	2.053	0.918	(−4.237, 3.816)	PFUA	−1.346	0.338	0.000	(−2.01, −0.682)
Lead	0.142	0.226	0.532	(−0.302, 0.586)	Lead	−0.069	0.038	0.068	(−0.144, 0.005)
Cadmium	−2.031	0.768	0.008	(−3.538, −0.524)	Cadmium	−0.036	0.132	0.786	(−0.295, 0.223)
Mercury	0.082	0.187	0.660	(−0.285, 0.449)	Mercury	−0.013	0.031	0.670	(−0.073, 0.047)

**Table 4.** Posterior inclusion probabilities (PIPs) for PFAS and metal exposures in relation to six cognitive outcome measures.

A: Trial 1		B: Trial 2		C: Trial 3	
Variable	PIP	Variable	PIP	Variable	PIP
PFDE	0.1600	PFDE	0.0000	PFDE	0.0300
PFOA	0.0000	PFOA	0.0000	PFOA	0.0172
PFOS	0.2256	PFOS	0.0016	PFOS	0.1916
PFHxS	0.0000	PFHxS	0.0000	PFHxS	0.0000
PFNA	0.0000	PFNA	0.0000	PFNA	0.0000
PFUA	0.0184	PFUA	0.0000	PFUA	0.0056
Lead	0.0000	Lead	0.0104	Lead	0.0000
Cadmium	0.0112	Cadmium	0.0260	Cadmium	0.0000
Mercury	0.1992	Mercury	0.0000	Mercury	0.0000
D: Animal Fluency		E: Digit Symbol Test		F: Delayed Recall Test	
Variable	PIP	Variable	PIP	Variable	PIP
FDE	0.0000	PFDE	0.0000	PFDE	0.0176
PFOA	0.0692	PFOA	1.0000	PFOA	0.0124
PFOS	0.0132	PFOS	0.0000	PFOS	0.0020
PFHxS	0.0000	PFHxS	0.0000	PFHxS	0.0000
PFNA	0.0000	PFNA	0.0000	PFNA	0.0000
PFUA	0.0612	PFUA	0.0000	PFUA	0.0000
Lead	0.0000	Lead	0.0000	Lead	0.0000
Cadmium	0.0000	Cadmium	0.0176	Cadmium	0.0000
Mercury	0.0000	Mercury	0.0000	Mercury	0.0000

## Data Availability

The NHANES dataset is publicly available online, accessible at https://wwwn.cdc.gov/nchs/nhanes/continuousnhanes/overview.aspx?BeginYear=2011 (accessed on 1 May 2025).
